# Canagliflozin, a sodium glucose co-transporter 2 inhibitor, improves model-based indices of beta cell function in patients with type 2 diabetes

**DOI:** 10.1007/s00125-014-3196-x

**Published:** 2014-03-01

**Authors:** David Polidori, Andrea Mari, Ele Ferrannini

**Affiliations:** 1 Janssen Research & Development, LLC, 3210 Merryfield Row, San Diego, CA 92121 USA; 2 Institute of Biomedical Engineering, National Research Council, Padua, Italy; 3 Department of Clinical and Experimental Medicine, University of Pisa School of Medicine, Pisa, Italy

**Keywords:** Beta cell function, Canagliflozin, Insulin secretion, SGLT2, Sodium glucose co-transporter 2 inhibitor, Type 2 diabetes

## Abstract

**Aims/hypothesis:**

In rodent models of diabetes, treatment with sodium glucose co-transporter 2 (SGLT2) inhibitors improves beta cell function. This analysis assessed the effects of the SGLT2 inhibitor, canagliflozin, on model-based measures of beta cell function in patients with type 2 diabetes.

**Methods:**

Data from three Phase 3 studies were analysed, in which: (Study 1) canagliflozin 100 and 300 mg were compared with placebo as monotherapy for 26 weeks; (Study 2) canagliflozin 100 and 300 mg were compared with placebo as add-on to metformin + sulfonylurea for 26 weeks; or (Study 3) canagliflozin 300 mg was compared with sitagliptin 100 mg as add-on to metformin + sulfonylurea for 52 weeks. In each study, a subset of patients was given mixed-meal tolerance tests at baseline and study endpoint, and model-based beta cell function parameters were calculated from plasma glucose and C-peptide.

**Results:**

In Studies 1 and 2, both canagliflozin doses increased beta cell glucose sensitivity compared with placebo. Placebo-subtracted least squares mean (LSM) (SEM) changes were 23 (9) and 18 (9) pmol min^−1^ m^−2^ (mmol/l)^−1^ with canagliflozin 100 and 300 mg, respectively (*p* < 0.002, Study 1), and 16 (8) and 10 (9) pmol min^−1^ m^−2^ (mmol/l)^−1^ (*p* < 0.02, Study 2). In Study 3, beta cell glucose sensitivity was minimally affected, but the insulin secretion rate at 9 mmol/l glucose increased to similar degrees from baseline with canagliflozin and sitagliptin [LSM (SEM) changes 38 (8) and 28 (9) pmol min^−1^ m^−2^, respectively; *p* < 0.05 for both].

**Conclusions/interpretation:**

Treatment with canagliflozin for 6 to 12 months improved model-based measures of beta cell function in three separate Phase 3 studies.

*Trial registration*: Clinicaltrials.gov NCT01081834 (Study 1); NCT01106625 (Study 2); NCT01137812 (Study 3)

**Electronic supplementary material:**

The online version of this article (doi:10.1007/s00125-014-3196-x) contains peer-reviewed but unedited supplementary material, which is available to authorised users.

## Introduction

Defects in beta cell function, including reduced insulin secretion and reduced efficiency of proinsulin conversion to insulin, are key pathophysiological factors underlying the hyperglycaemia of patients with type 2 diabetes mellitus [1, 2]. Declining beta cell function is a major contributing factor to the progressive nature of type 2 diabetes, with many patients eventually requiring insulin therapy to achieve and maintain glycaemic control [3, 4]. Glucose-lowering agents, which in addition to lowering plasma glucose levels, can improve beta cell function or slow the progression of beta cell dysfunction, may be useful for the long-term management of type 2 diabetes [1].

Pharmacological inhibition of the sodium glucose co-transporter 2 (SGLT2) is a novel approach to lowering plasma glucose in individuals with hyperglycaemia. SGLT2 inhibitors block renal glucose reabsorption and lower the renal threshold for glucose, thereby markedly increasing urinary glucose excretion (UGE) [5, 6]. Studies in animals have shown that beta cell function is restored when normoglycaemia is achieved by treatment with SGLT2 inhibitors [7–10]. The improvements in beta cell function observed in animal models are believed to be secondary to the improved glucose control, rather than due to direct effects of SGLT2 inhibitors on beta cells.

Canagliflozin is an SGLT2 inhibitor developed for the treatment of patients with type 2 diabetes [11–18]. In Phase 3 clinical studies, canagliflozin 100 and 300 mg provided significant improvements in glycaemic control in adult patients with type 2 diabetes, both as monotherapy and as add-on therapy to various background diabetes treatments [11–13, 15–17]. In addition, three clinical studies have evaluated the effects of canagliflozin treatment on measures of beta cell function in patients with type 2 diabetes. The first (Study 1) [16] was a study of canagliflozin 100 and 300 mg monotherapy compared with placebo at 26 weeks. The second (Study 2) [18] studied canagliflozin 100 and 300 mg as add-on therapy to metformin plus a sulfonylurea compared with placebo at 26 weeks; and the third (Study 3) [15] studied canagliflozin 300 mg as add-on therapy to metformin plus a sulfonylurea compared with sitagliptin 100 mg at 52 weeks. In these studies, canagliflozin treatment was generally associated with improvements in surrogate measures of beta cell function, including HOMA-2-derived beta cell function (HOMA2-%B), the proinsulin:C-peptide ratio and the C-peptide AUC:glucose AUC ratio. The current report presents further data from these three studies characterising the effects of canagliflozin treatment on additional indices of beta cell function obtained from the model-based analysis of plasma glucose and C-peptide responses to mixed-meal tolerance tests (MMTTs).

## Methods

### Patients and study design

Study 1 (canagliflozin monotherapy; ClinicalTrials.gov identifier NCT01081834) [16] and Study 2 (canagliflozin as add-on to metformin and sulfonylurea; ClinicalTrials.gov identifier NCT01106625) [18] were both randomised, double-blind, placebo-controlled, Phase 3 studies with a 26 week core treatment period followed by a 26 week extension period; findings on beta cell function from the core treatment periods are reported here. Study 3 (ClinicalTrials.gov identifier NCT01137812) [15] was a 52 week, randomised, double-blind, active-controlled, Phase 3 study comparing canagliflozin 300 mg with sitagliptin 100 mg as add-on therapy to metformin plus sulfonylurea. Details of study design, including patient inclusion and exclusion criteria, have been previously reported [15, 16, 18]. For all three studies, key aspects of study design, eligibility criteria and patient populations are summarised in Table 1.

**Table 1:** Summary of study designs and patient populations

Study characteristics				Eligibility criteria				mITT population (FS-MMTT subset), *n*			
Study	Treatment	Comparator	Duration^a^	Age^c^	HbA_1c_^d^	FPG^e^	eGFR^f^	PBO	CANA 100	CANA 300	SITA 100
1	Monotherapy [16]	PBO	26^b^	18–80	7.0–10.0 (53–86)	<15.0 (270)	≥50	192 (76)	195 (79)	197 (80)	NA
2	Add-on to MET + SU [18]	PBO	26^b^	18–80	7.0–10.5 (53–91)	<15.0 (270)	≥55^g^	156 (55)	157 (57)	156 (56)	NA
3	Add-on to MET + SU [15]	SITA	52	≥18	7.0–10.5 (53–91)	<16.7 (300)	≥55^g^	NA	NA	377 (125)	378 (124)

^a^In weeks

^b^Results from the 26-week core treatment period are reported here; study also included a 26-week extension period

^c^In years

^d^In % (mmol/mol)

^e^In mmol/l (mg/dl)

^f^In ml min^−1^ (1.73 m^2^)^−1^

^g^eGFR ≥60 ml min^−1^ (1.73 m^2^)^−1^ if required, based on the local MET label

CANA 100, canagliflozin 100 mg; CANA 300, canagliflozin 300 mg; eGFR, estimated GFR; MET, metformin; NA, not applicable; PBO, placebo; SITA 100, sitagliptin 100 mg; SU, sulfonylurea

These studies were conducted in accordance with the ethical principles detailed in the Declaration of Helsinki, and are consistent with good clinical practice and applicable regulatory requirements. Approval of the study protocols and amendments was obtained from institutional review boards and independent ethics committees of the participating centres. All patients provided informed written consent prior to participation.

### Randomisation and study treatments

In Studies 1 and 2, patients were randomly assigned to receive daily oral doses of canagliflozin 100 or 300 mg or placebo (1:1:1); randomisation was stratified according to whether individuals had participated in the frequently sampled (FS)-MMTT (Studies 1 and 2), and whether they were taking glucose-lowering agents at screening (Study 1) or had begun a glucose-lowering agent adjustment period (Study 2). In Study 3, patients were randomly assigned to receive daily oral doses of canagliflozin 300 mg or sitagliptin 100 mg (1:1). Randomisation was stratified according to whether individuals had participated in the FS-MMTT and whether their pre-randomisation HbA_1c_ value was ≥9.0% (75 mmol/mol).

In Studies 1 and 2, glycaemic rescue therapy with metformin (Study 1) or insulin (Study 2) was initiated at fasting plasma glucose (FPG) >15.0 mmol/l (270 mg/dl) from Day 1 to Week 6, >13.3 mmol/l (240 mg/dl) from Week 6 to Week 12 and >11.1 mmol/l (200 mg/dl) from Week 12 to Week 26. No glycaemic rescue therapy was provided in Study 3; patients meeting pre-specified glycaemic criteria (same as for Studies 1 and 2 through to Week 26, HbA_1c_ >8.0% [64 mmol/mol] after Week 26) were discontinued.

### Endpoints and assessments

A subset of patients from each study underwent an FS-MMTT on Day 1 and at Week 26 in Studies 1 and 2, and at Week 52 in Study 3. For the Day 1 test, patients took their first dose of study medication after blood samples for the MMTT were collected. For the FS-MMTT at Week 26 (Studies 1 and 2) or Week 52 (Study 3), the last dose of study medication was administered approximately 30 min before the start of the standard meal. Patients fasted for at least 8 h before the start of the FS-MMTT. The standardised meal was given between 07:00 and 10:00 hours, and consisted of two servings of liquid supplement containing approximately 100 g of total carbohydrates and approximately 2930 kJ (700 kcal). Water or non-caffeinated energy-free beverages could be consumed without restriction. The entire meal was to be completed within a 15 min period.

During the FS-MMTT, blood samples were collected for the measurement of glucose, insulin and C-peptide 15 min before and immediately prior to the start of the meal, and at 30, 60, 90, 120 and 180 min after the meal. Patients were asked to empty their bladders immediately prior to collection of the first blood sample. Urine was then collected over the interval from 15 min before the meal through to 180 min after the meal for the measurement of UGE. In Study 3, mean plasma glucose during the 3 h postprandial period (MPG_0–3 h_) was calculated as the AUC for the plasma glucose profile (calculated by the trapezoid rule) divided by the 3 h time interval.

### Modelling analysis

Beta cell function was assessed from the FS-MMTT using a model that describes the relationship between insulin secretion and glucose concentration, and which has previously been described in detail [19, 20]. The model expresses insulin secretion (in pmol min^−1^ m^−2^) as the sum of two components. The first of these components consists of a dose–response function relating the insulin secretion rate and the absolute glucose concentration at any time point during the MMTT. Characteristic parameters of the dose–response function are the mean slope over the observed glucose range, denoted as beta cell glucose sensitivity, and the insulin secretion rate (ISR) at a fixed glucose level of 9 mmol/l (i.e. ISR at 9 mmol/l glucose). The dose–response function is modulated by a potentiation factor encompassing several potentiating mechanisms (e.g. prolonged exposure to hyperglycaemia, non-glucose substrates, gastrointestinal hormones and neural modulation), which is set to be a positive function of time and is constrained to average unity during the experiment. The second insulin secretion component, termed the derivative component, represents the dependence of insulin secretion on the rate of change of glucose concentration and is determined by a single parameter, denoted as rate sensitivity, that is related to early insulin release [21].

The model parameters were estimated from glucose and C-peptide concentrations by regularised least squares, as previously described [19]. The regularisation process involves a choice of smoothing factors. These were selected to obtain glucose and C-peptide model residuals with SDs close to the expected measurement error (∼1% for glucose, ∼4% for C-peptide). The ISR was calculated from the model every 5 min. The integral of insulin secretion during the 3 h MMTT was denoted as total insulin secretion, and insulin clearance was calculated by dividing total insulin secretion by plasma insulin AUC. Insulin clearance was calculated only for participants with at least five measurements of plasma insulin during the MMTT.

Insulin sensitivity was estimated from the MMTT data using the oral glucose insulin sensitivity (OGIS) index as previously described [22, 23]. However, because this index does not account for the insulin-independent glucose lowering occurring due to UGE in canagliflozin-treated participants, the OGIS index will tend to overestimate improvements in insulin sensitivity in canagliflozin-treated participants. To account for this, UGE-corrected OGIS values (OGIS_c_) were calculated by subtracting renal glucose clearance (calculated as UGE divided by plasma glucose AUC during the MMTT) from the OGIS value to provide a more appropriate index of insulin sensitivity for assessing the effects of canagliflozin treatment.

### Statistical analyses

Results for all model parameters are presented for all patients in the modified intent-to-treat (mITT) analysis set (i.e. all randomised patients who received ≥1 dose of study drug) with MMTT data at baseline and study endpoint. Statistical analyses for ISR at 9 mmol/l glucose, beta cell glucose sensitivity, OGIS, UGE, total insulin secretion and insulin clearance were performed using ANCOVA models, with the model parameter as the response variable, the baseline parameter value and baseline glycaemic control (binary value assessing whether baseline HbA_1c_ was <9.0% [75 mmol/mol]) as covariates, and treatment as the experimental factor. The distribution of values for beta cell glucose sensitivity and the OGIS measures were better approximated by a log-normal than a normal distribution, so analyses of these parameters were performed using both original and log-transformed data; for clarity, the results (change in least squares mean [ΔLSM] and SEM) are presented in the original scale, but statistical significance testing was performed using the log-transformed values. The distribution of values for the rate sensitivity parameter was highly non-normal, so between-group comparisons were assessed using Kruskal–Wallis tests. In Study 3, similar ANCOVA models to those used for ISR at 9 mmol/l glucose were used for comparison of FPG, MPG_0–3 h_ and peak postprandial plasma glucose concentrations. Differences between treatments were considered statistically significant at *p* < 0.05.

## Results

### Patient disposition and baseline characteristics

**Fig. 1:**
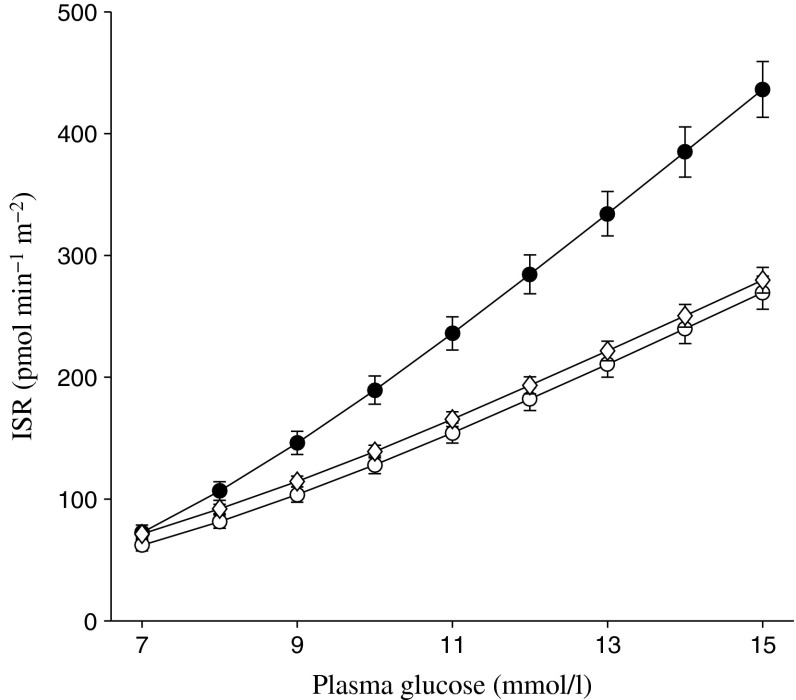
Baseline (pretreatment) relationship between insulin secretion and plasma glucose concentrations (Studies 1 to 3). Black circles, untreated (Study 1; *n* = 193); white circles, metformin + sulfonylurea (Study 2; *n* = 163); white diamonds, metformin + sulfonylurea (Study 3; *n* = 234). Values are mean ± SEM and include all patients studied at the baseline visit in each study

**Fig. 2:**
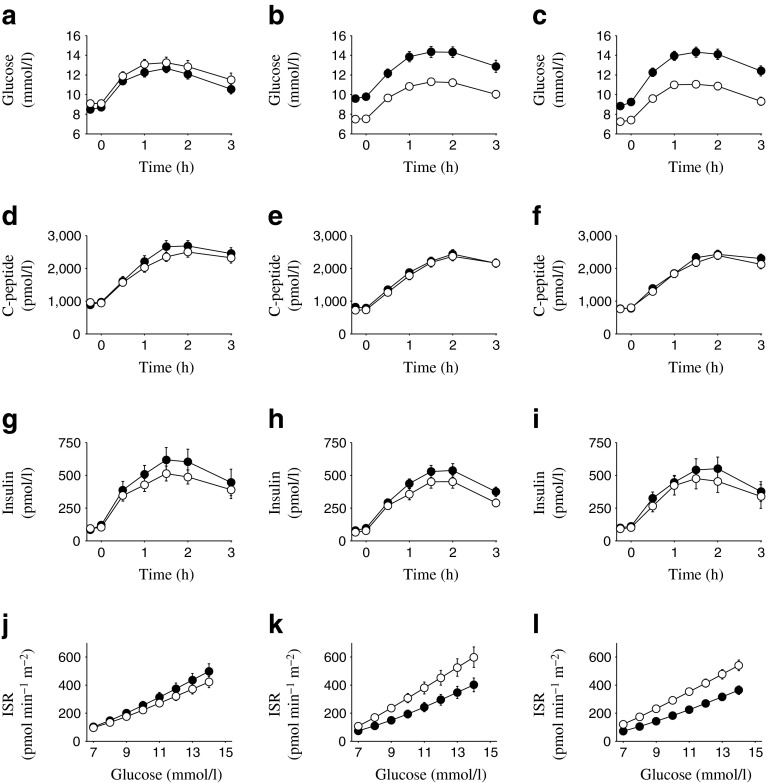
(**a–c**) Plasma glucose, (**d–f**) C-peptide and (**g–i**) insulin concentrations, and (**j–l**) ISR per plasma glucose values in Study 1. Black circles, baseline; white circles, Week 26. Values are mean ± SEM for placebo (**a**, **d**, **g**, **j**), canagliflozin 100 mg (**b**, **e**, **h**, **k**) and canagliflozin 300 mg (**c**, **f**, **i**, **l**)

**Fig. 3:**
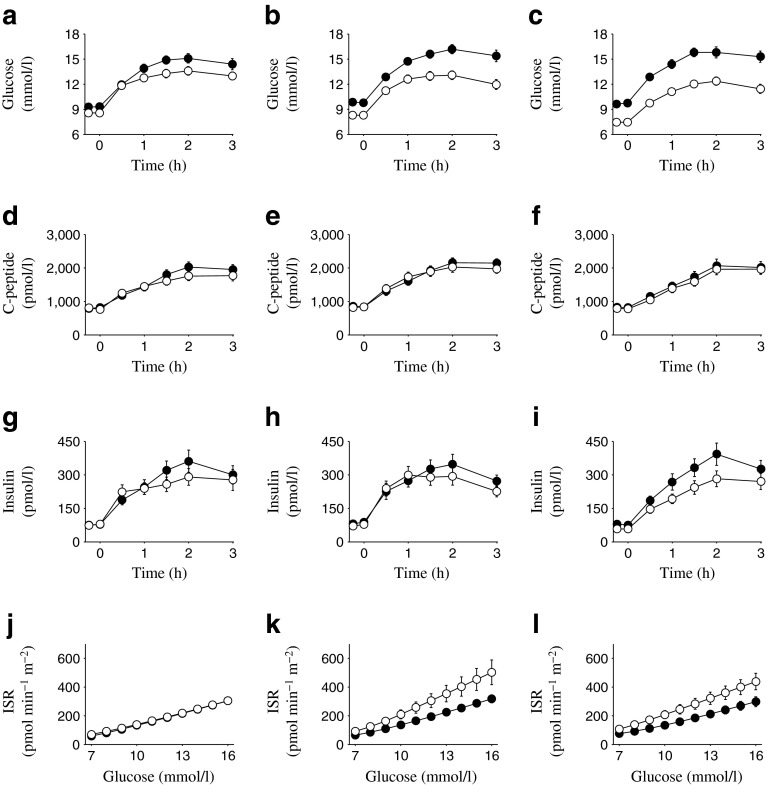
(**a–c**) Plasma glucose, (**d–f**) C-peptide and (**g–i**) insulin concentrations, and (**j–l**) ISR per plasma glucose values in Study 2. Black circles, baseline; white circles, Week 26. Values are mean ± SEM for placebo (**a**, **d**, **g**, **j**), canagliflozin 100 mg (**b**, **e**, **h**, **k**) and canagliflozin 300 mg (**c**, **f**, **i**, **l**)

**Fig. 4:**
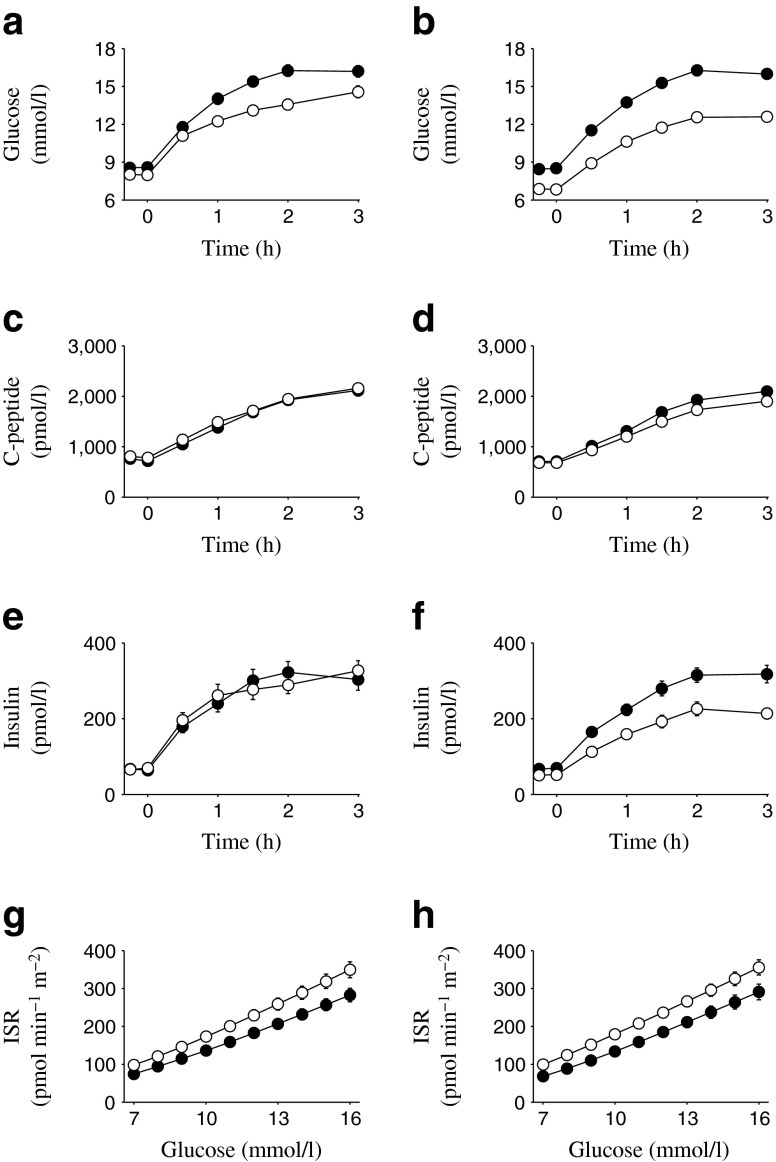
(**a**, **b**) Plasma glucose, (**c**, **d**) C-peptide and (**e**, **f**) insulin concentrations, and (**g**, **h**) ISR per plasma glucose in Study 3. Black circles, baseline; white circles, Week 52. Values are mean ± SEM for sitagliptin 100 mg (**a**, **c**, **e**, **g**) and canagliflozin 300 mg (**b**, **d**, **f**, **h**)

Baseline demographic and disease characteristics for patients in the FS-MMTT subpopulations are summarised in Table 2. The characteristics of these patients are similar to those observed in the overall population in each study [15, 16, 18]. The baseline relationship between ISR and plasma glucose in each of the three studies is shown in Fig. 1. Consistent with the well established progressive nature of beta cell failure in type 2 diabetes, baseline beta cell function was better in patients in Study 1 than in the patients with longer duration of diabetes evaluated in Studies 2 and 3; this is shown by the steeper slope for the relationship between ISR and plasma glucose in Study 1 compared with Studies 2 and 3. Within each study, baseline plasma glucose, insulin and C-peptide profiles, and the corresponding ISR–plasma glucose relationships at baseline were generally similar between the different treatment groups, with the exception of the placebo group in Study 1, which had modestly lower mean plasma glucose concentrations and modestly higher plasma C-peptide concentrations than the two canagliflozin groups, leading to a shift in the mean relationship between baseline ISR and plasma glucose concentration in the placebo compared with the canagliflozin groups (Figs 2, 3, 4).

**Table 2:** Baseline demographic and disease characteristics for the FS-MMTT subpopulations

Characteristic	Study 1				Study 2				Study 3		
	PBO	CANA 100	CANA 300	Total	PBO	CANA 100	CANA 300	Total	CANA 300	SITA 100	Total
*n*	61	64	68	193	53	56	54	163	117	117	234
Sex, *n* (%)^a^											
Men	29 (48)	19 (30)	33 (49)	81 (42)	30 (57)	28 (50)	26 (48)	84 (52)	66 (56)	64 (55)	130 (56)
Women	32 (53)	45 (70)	35 (52)	112 (58)	23 (43)	28 (50)	28 (52)	79 (49)	51 (44)	53 (45)	104 (44)
Age, years	58 ± 12	56 ± 10	55 ± 12	56 ± 11	56 ± 9	57 ± 11	55 ± 8	56 ± 9	58 ± 9	57 ± 8	57 ± 9
Race, *n* (%)^a^											
White	49 (80)	46 (72)	56 (82)	151 (78)	43 (81)	51 (91)	42 (78)	136 (83)	79 (68)	80 (68)	159 (68)
Black/African-American	4 (7)	10 (16)	6 (9)	20 (10)	4 (8)	0	6 (11)	10 (6)	20 (17)	15 (13)	35 (15)
Asian	1 (2)	0	1 (2)	2 (1)	1 (2)	1 (2)	0	2 (1)	7 (6)	4 (3)	11 (5)
Other^b^	7 (12)	8 (13)	5 (7)	20 (10)	5 (9)	4 (7)	6 (11)	15 (9)	11 (9)	18 (15)	29 (12)
HbA_1c_, %	7.7 ± 0.9	8.0 ± 0.9	7.9 ± 0.9	7.9 ± 0.9	8.1 ± 0.8	8.2 ± 1.0	8.3 ± 1.1	8.2 ± 1.0	8.0 ± 0.9	8.1 ± 0.9	8.1 ± 0.9
HbA_1c_, mmol/mol	61 ± 9.8	64 ± 9.8	63 ± 9.8	63 ± 9.8	65 ± 8.7	66 ± 10.9	67 ± 12.0	66 ± 10.9	64 ± 9.8	65 ± 9.8	65 ± 9.8
FPG, mmol/l	9.0 ± 2.1	9.7 ± 2.3	9.3 ± 2.1	9.3 ± 2.2	9.7 ± 2.3	10.0 ± 2.4	9.7 ± 2.4	9.8 ± 2.4	8.8 ± 2.2	8.8 ± 2.4	8.8 ± 2.3
Body weight, kg	93 ± 17	87 ± 20	90 ± 22	90 ± 20	90 ± 22	93 ± 21	92 ± 19	92 ± 21	84 ± 20	88 ± 21	86 ± 21
Waist circumference, cm	110 ± 13	104 ± 13	106 ± 15	107 ± 14	105 ± 15	109 ± 16	108 ± 14	108 ± 15	104 ± 13	106 ± 13	105 ± 13
Duration of diabetes, years	4.4 ± 4.5	5.5 ± 4.3	5.3 ± 5.5	5.1 ± 4.8	10.5 ± 6.7	9.6 ± 6.1	8.9 ± 5.4	9.7 ± 6.1	9.9 ± 6.4	10.1 ± 6.8	10.0 ± 6.6

Data are mean ± SD unless otherwise indicated

^a^Percentages may not total 100% due to rounding

^b^Includes American Indian or Alaska Native, Native Hawaiian or other Pacific Islander, multiple, other or not reported

PBO, placebo; CANA 100, canagliflozin 100 mg; CANA 300, canagliflozin 300 mg; SITA 100, sitagliptin 100 mg

### Effects of canagliflozin treatment on HbA_1c_, body weight and waist circumference

As previously reported [15, 16, 18], in each of the three studies canagliflozin treatment reduced HbA_1c_, body weight and waist circumference. Greater reductions were observed for all three of these measures with canagliflozin treatment than with placebo (Studies 1 and 2) or sitagliptin (Study 3). The changes in these measures in the subset of patients with MMTT data (Table 3) were similar to the changes observed in the complete studies.

**Table 3:** Changes in HbA_1c_, body weight and waist circumference in FS-MMTT subpopulations

Parameters	Study 1			Study 2			Study 3	
	PBO	CANA 100	CANA 300	PBO	CANA 100	CANA 300	CANA 300	SITA 100
HbA_1c_, *n*	39	51	50	33	43	36	73	61
Baseline, %	7.45 (0.69)	7.92 (0.85)	7.84 (0.88)	7.98 (0.66)	8.00 (0.87)	8.27 (1.04)	8.00 (0.90)	7.97 (0.84)
Endpoint, %^a^	7.55 (1.02)	7.12 (0.77)	6.79 (0.72)	7.78 (0.91)	7.01 (0.80)	6.98 (0.97)	6.85 (0.68)	7.10 (0.83)
ΔLSM (95% CI), %^b^	–	−0.68 (−0.98, −0.37)	−0.99 (−1.30, −0.69)	–	−0.73 (−1.12, −0.34)	−0.86 (−1.27, −0.45)	−0.26 (−0.5, −0.02)	–
Baseline, mmol/mol	57.9 (7.6)	63.0 (9.3)	62.2 (9.6)	63.7 (7.2)	64.0 (9.5)	66.9 (11.4)	64.0 (9.9)	63.6 (9.2)
Endpoint, mmol/mol^a^	59.0 (11.1)	54.3 (8.5)	50.7 (7.9)	61.5 (9.9)	53.2 (8.7)	52.7 (10.6)	51.4 (7.4)	54.1 (9.0)
ΔLSM (95% CI), mmol/mol^b^	–	−7.4 (−10.7, −4.1)	−10.8 (−14.2, −7.6)	–	−8.0 (−12.3, −3.7)	−9.4 (−13.8, −4.9)	−2.8 (−5.5, −0.2)	–
Body weight, *n*	39	51	50	33	43	36	73	61
Baseline, kg	95 (16)	88 (20)	89 (22)	88 (22)	91 (19)	94 (18)	83 (20)	86 (21)
Endpoint, kg^a^	95 (16)	85 (21)	85 (21)	88 (22)	89 (19)	91 (18)	80 (19)	86 (20)
ΔLSM (95% CI), % change^b^	–	−3.6 (−4.9, −2.3)	−4.2 (−5.4, −2.9)	–	−1.9 (−3.1, −0.6)	−2.2 (−3.5, −0.9)	−3.6 (−4.9, −2.3)	–
Waist circumference, *n*	38	50	49	33	43	36	73	61
Baseline, cm	111 (12)	105 (13)	106 (14)	104 (15)	108 (14)	108 (13)	103 (13)	105 (12)
Endpoint, cm^a^	111 (11)	103 (13)	104 (14)	103 (15)	104 (15)	105 (12)	102 (13)	105 (12)
ΔLSM (95% CI), cm^b^	–	−1.5 (−3.2, 0.3)	−1.8 (−3.5, −0.01)	–	−3.5 (−6.3, −0.7)	−2.3 (−5.2, 0.6)	−1.4 (−2.9, 0.02)	–

Data are mean (SD) unless otherwise indicated

^a^Week 26 for Studies 1 and 2; Week 52 for Study 3

^b^ΔLSM is the PBO-subtracted LSM change from baseline for Studies 1 and 2, and the SITA-subtracted LSM change in Study 3

PBO, placebo; CANA 100, canagliflozin 100 mg; CANA 300, canagliflozin 300 mg; SITA 100, sitagliptin 100 mg

### Effects of canagliflozin treatment on UGE, fasting and postprandial glucose, and insulin and C-peptide

In each of the three studies, canagliflozin treatment increased UGE (Table 4), resulting in reduced fasting and postprandial glucose concentrations (Figs 2, 3, 4). Despite the decreases in plasma glucose concentrations observed with canagliflozin treatment, C-peptide concentrations were generally similar at baseline and the study endpoint (Week 26 for Studies 1 and 2, Week 52 for Study 3) in canagliflozin-treated patients, whereas in the same patients plasma insulin concentrations tended to be modestly lower at the study endpoint relative to baseline (Figs 2, 3, 4). In the sitagliptin comparator study (Study 3), greater reductions in fasting and postprandial glucose were seen with canagliflozin 300 mg than with sitagliptin 100 mg (electronic supplementary material [ESM] Table 1).

**Table 4:** UGE measured during the MMTT (0–3 h)

Urine glucose, g	Study 1			Study 2			Study 3	
	PBO	CANA 100	CANA 300	PBO	CANA 100	CANA 300	CANA 300	SITA 100
Baseline, *n*	46	46	55	45	52	47	111	107
Mean (SD)	3.9 (7.4)	4.9 (7.0)	4.1 (5.6)	3.0 (4.8)	3.4 (5.6)	3.9 (4.1)	4.4 (5.6)	3.7 (4.2)
Endpoint, *n* ^a^	34	53	48	28	42	33	73	51
Mean (SD)	3.9 (8.8)	16.4 (15.1)	18.8 (19.3)	4.4 (12.4)	13.6 (14.9)	19.7 (23.3)	21.4 (34.8)	3.2 (4.8)
ΔLSM (95% CI)^b^	–	11.3 (3.7, 19.0)	14.7 (7.2, 22.3)	–	9.2 (0.8, 17.6)	15.8 (6.8, 24.7)	17.7 (6.8, 28.6)	–

^a^Week 26 for Studies 1 and 2; Week 52 for Study 3

^b^ΔLSM is the PBO-subtracted LSM change from baseline (95% CI) for Studies 1 and 2, and the SITA-subtracted LSM change in Study 3

CANA 100, canagliflozin 100 mg; CANA 300, canagliflozin 300 mg; PBO, placebo; SITA 100, sitagliptin 100 mg

### Measures of beta cell function and insulin sensitivity

In Study 1, both doses of canagliflozin led to an upward shift in the relationship between ISR and plasma glucose, as well as an increased slope (Fig. 2j–l), whereas a slight downward shift was observed with placebo. The model-assessed parameters for ISR at 9 mmol/l glucose and beta cell glucose sensitivity were significantly increased with both doses of canagliflozin (Table 5). Changes in the rate sensitivity parameter varied considerably between patients, with no significant effect of canagliflozin compared with placebo. Total insulin secretion during the MMTT was not statistically significantly different between the canagliflozin and placebo groups. Insulin clearance was increased by approximately 15% with both doses of canagliflozin compared with placebo (ESM Table 2).

**Table 5:** Beta cell function and insulin sensitivity parameters

Parameter	Study 1			Study 2			Study 3	
	PBO	CANA 100	CANA 300	PBO	CANA 100	CANA 300	CANA 300	SITA 100
*n*	39	51	50	33	43	36	73	61
ISR^a^								
Baseline	200 (143)	150 (170)	143 (109)	106 (59)	109 (94)	122 (91)	111 (59)	115 (62)
Endpoint^b^	175 (117)	237 (171)	232 (124)	114 (63)	163 (62)	172 (144)	152 (66)	146 (87)
ΔLSM (SEM)^c^	–	95 (23)	96 (24)	–	40 (25)	47 (26)	38 (8)	28 (9)
*p* value^d^	–	<0.0001	<0.0001	–	0.10	0.07	0.40	–
Beta cell GS^e^								
Baseline	58 (39)	52 (38)	45 (23)	28 (15)	30 (18)	27 (20)	26 (20)	25 (16)
Endpoint^b^	50 (33)	68 (65)	59 (30)	27 (16)	46 (60)	36 (60)	28 (15)	29 (15)
ΔLSM (SEM)^c^	–	23 (9)	18 (9)	–	16 (8)	10 (9)	1 (1)	2 (2)
*p* value^d^	–	0.0007	0.002	–	0.02	0.02	0.95	–
Rate sensitivity^f^								
Baseline	468 (550)	566 (810)	471 (532)	401 (411)	376 (521)	218 (358)	246 (354)	270 (406)
Endpoint^b^	459 (518)	412 (537)	324 (548)	519 (574)	364 (494)	154 (323)	265 (474)	256 (508)
ΔMean (SEM)^g^	−9 (78)	−154 (120)	−147 (101)	118 (85)	−12 (114)	−64 (72)	19 (68)	−14 (82)
*p* value^d^	–	0.55	0.17	–	0.20	0.51	0.40	–
Total insulin secretion^h^								
Baseline	58 (25)	52 (19)	54 (19)	43 (18)	45 (20)	42 (21)	42 (16)	43 (17)
Endpoint^b^	53 (20)	50 (19)	52 (19)	40 (16)	45 (21)	41 (20)	38 (15)	44 (14)
ΔLSM (SEM)^c^	–	2.6 (2.4)	2.5 (2.4)	–	3.4 (2.6)	1.8 (2.7)	−4.8 (1.7)	–
*p* value^d^	–	0.29	0.29	–	0.20	0.51	0.005	–
OGIS^i^								
*n* ^j^	32	46	43	32	37	32	66	57
Baseline	264 (45)	250 (57)	258 (44)	272 (46)	250 (38)	270 (60)	292 (58)	293 (81)
Endpoint^b^	265 (63)	304 (58)	311 (57)	298 (89)	298 (75)	325 (68)	356 (77)	305 (64)
ΔLSM (SEM)^c^	–	50 (12)	52 (12)	–	11 (18)	28 (19)	51(12)	–
*p* value^d^	–	<0.0001	<0.0001	–	0.46	0.06	<0.0001	–
OGIS_c_^j^								
*n* ^k^	32	44	38	32	33	26	64	57
Baseline	263 (45)	244 (58)	258 (45)	269 (48)	249 (40)	269 (65)	286 (59)	290 (82)
Endpoint^b^	263 (63)	279 (60)	289 (61)	297 (89)	286 (81)	304 (69)	331 (77)	302 (65)
ΔLSM (SEM)^c^	–	28 (13)	31 (13)	–	0.4 (19)	8 (20)	30 (12)	–
*p* value^d^	–	0.01	0.01	–	0.98	0.54	0.02	–

Data are mean (SD) unless otherwise indicated

^a^In pmol min^−1^ m^−2^ at 9 mmol/l glucose

^b^Week 26 for Studies 1 and 2; Week 52 for Study 3

^c^ΔLSM is the PBO-subtracted LSM change from baseline for Studies 1 and 2 and the LSM change from baseline for Study 3. For glucose sensitivity, ΔLSM values are reported for the untransformed variables, but statistical testing was performed on log-transformed values

^d^ *p* values vs PBO for Studies 1 and 2, and vs SITA for Study 3

^e^In pmol min^−1^ m^−2^ (mmol/l)^−1^

^f^In pmol m^−2^ (mmol/l)^−1^

^g^ΔMean is the mean change from baseline

^h^In pmol/m^2^

^i^In ml min^−1^ m^−2^; not corrected for UGE

^j^In ml min^−1^ m^−2^

^k^The number of patients with OGIS values is smaller than the number of patients with the other measures, as some patients had insufficient insulin and/or UGE measurements to perform the OGIS calculations

CANA 100, canagliflozin 100 mg; CANA 300, canagliflozin 300 mg; GS, glucose sensitivity; PBO, placebo; SITA 100, sitagliptin 100 mg

The effects of canagliflozin on beta cell function observed in Study 2 were generally similar to those seen in Study 1, with both doses of canagliflozin leading to an upward shift and steepening of the curve expressing the relationship between ISR and plasma glucose. No change was observed with placebo (Fig. 3j–l). In Study 2, the placebo-subtracted LSM increases in ISR at 9 mmol/l glucose and in beta cell glucose sensitivity were smaller than those observed in Study 1 (Table 5), with the former (ISR at 9 mmol/l) not quite achieving statistical significance (*p* = 0.10 for canagliflozin 100 mg, *p =* 0.07 for canagliflozin 300 mg). Insulin clearance was increased with both doses of canagliflozin compared with placebo, although the increase observed with the 100 mg dose did not reach statistical significance (*p* = 0.07) (ESM Table 2); the increase in insulin clearance observed with the 300 mg dose compared with placebo was approximately 24% (*p* < 0.0001).

In Study 3, treatment with sitagliptin 100 mg and canagliflozin 300 mg produced similar upward shifts in the relationship between ISR and plasma glucose (Fig. 4g, h). Increases from baseline in ISR at 9 mmol/l glucose were observed with sitagliptin (28 pmol min^−1^ m^−2^) and canagliflozin (38 pmol min^−1^ m^−2^; *p* < 0.05 vs baseline for both) (Table 5), with the increase observed with canagliflozin being similar to that observed in Study 2. However, the difference between canagliflozin and sitagliptin was not statistically significant (*p* = 0.4). Only minimal changes in beta cell glucose sensitivity were observed in either treatment group (1–2 pmol min^−1^ m^−2^ [mmol/l]^−1^) in this study, while no differences in rate sensitivity were observed between groups. Consistent with the greater reductions in plasma glucose concentrations in the canagliflozin group, total insulin secretion was reduced with canagliflozin treatment compared with sitagliptin (*p =* 0.005) (Table 5). Insulin clearance was increased by approximately 30% with canagliflozin 300 mg, whereas no change was observed with sitagliptin treatment (ESM Table 2).

Insulin sensitivity, as assessed by the UGE-corrected OGIS values, also improved with canagliflozin treatment (Table 5), with mean values of OGIS_c_ increasing by approximately 15% in canagliflozin-treated patients (although the differences were not significantly different from placebo in Study 2). As expected, the correction made to OGIS to account for UGE led to smaller increases in the index than those obtained using the uncorrected values.

## Discussion

Progressive loss of beta cell function is a hallmark of type 2 diabetes and contributes to the progressive nature of the disease [1]. Several treatments for type 2 diabetes (e.g. sulfonylureas, meglitinides, glucagon-like peptide-1 [GLP-1] receptor agonists and dipeptidyl peptidase-4 [DPP-4] inhibitors) directly stimulate beta cells to increase insulin release. Using the same methods as in this paper, the DPP-4 inhibitors vildagliptin and sitagliptin [24, 25] and the GLP-1 receptor agonists liraglutide and exenatide [26, 27] have all been previously shown to improve indices of beta cell function.

SGLT2 inhibitors lower plasma glucose concentrations through a novel mechanism of action that does not directly affect insulin secretion or insulin sensitivity. SGLT2 inhibition reduces the renal threshold for glucose excretion, leading to increased UGE and thereby lowering plasma glucose concentrations [5, 6]. In several preclinical studies, the reversal of hyperglycaemia by treating hyperglycaemic rodents with SGLT2 inhibitors led to improved beta cell function and mass [7–10]. The present studies demonstrate that sustained treatment with the SGLT2 inhibitor canagliflozin for 6 to 12 months improved fasting and postprandial measures of beta cell function in humans. The plasma glucose and HbA_1c_ reductions observed in the FS-MMTT patient subsets are consistent with the corresponding reductions observed in the overall populations of the three studies [15, 16, 18]. In Study 3, the improvements in beta cell function obtained from the FS-MMTT analysis were similar with canagliflozin and sitagliptin treatment, even though sitagliptin has direct effects on beta cells through the elevation of GLP-1 and glucose-dependent insulinotropic peptide, whereas canagliflozin is not believed to have any direct effects on beta cells. Although both treatments caused similar increases in ISR relative to glucose during the MMTT, the fasting proinsulin:C-peptide ratio was decreased with canagliflozin treatment compared with sitagliptin treatment [15], suggesting that the treatments have different effects on proinsulin processing [28]. The improvements in measures of beta cell function observed in canagliflozin-treated patients are believed to be secondary to improvements in plasma glucose control, rather than direct effects of canagliflozin, as no changes in measures of insulin secretion have been observed in normoglycaemic participants treated with canagliflozin. For example, no notable changes in 24 h plasma insulin profiles were observed in healthy participants treated with canagliflozin [29] and, in a separate study with an MMTT in healthy participants treated with canagliflozin 300 mg or placebo, no differences in the relationship between ISR and plasma glucose were observed between the canagliflozin and placebo treatment groups [5].

Canagliflozin treatment generally led to greater reductions in plasma insulin concentrations compared with C-peptide concentrations, suggesting that canagliflozin treatment altered insulin and/or C-peptide clearance, with the calculated increases in insulin clearance with canagliflozin treatment generally ranging from approximately 15% to 30%. The ISRs and the corresponding insulin clearance values calculated in this study were obtained using the assumption that canagliflozin treatment did not alter C-peptide kinetics. Because the kidney is the primary site of C-peptide clearance and canagliflozin acts directly on the kidney, a separate study was performed to assess whether canagliflozin treatment alters C-peptide clearance. This study showed that canagliflozin had only minimal effects on C-peptide clearance (<4%), confirming that the deconvolution procedure used to estimate ISR is appropriate in canagliflozin-treated patients [30]. Thus, it is likely that insulin clearance, which is known to be highly variable due to large and variable first-pass hepatic extraction [31], was increased with sustained canagliflozin treatment.

In conclusion, the results from three separate Phase 3 studies demonstrate that sustained treatment with canagliflozin for 6 to 12 months improves measures of beta cell function. It is now important to obtain data from longer term studies to assess whether treatment with canagliflozin can help slow the progressive decline of beta cell function over a longer period.

## Electronic supplementary material

Below is the link to the electronic supplementary material.

ESM Table 1 (PDF 33 kb)

ESM Table 2 (PDF 31 kb)

## Abbreviations

DPP-4: Dipeptidyl peptidase-4
FPG: Fasting plasma glucose
FS: Frequently sampled
GLP-1: Glucagon-like peptide-1
ISR: Insulin secretion rate
LSM: Least squares mean
mITT: Modified intent-to-treat
MMTT: Mixed-meal tolerance test
MPG_0–3 h_: Mean plasma glucose during the 3 h postprandial period
OGIS: Oral glucose insulin sensitivity
OGIS_c_: UGE-corrected OGIS values
SGLT2: Sodium glucose co-transporter 2
UGE: Urinary glucose excretion
