# Supplementary material for: Canagliflozin, a sodium glucose co-transporter 2 inhibitor, improves model-based indices of beta cell function in patients with type 2 diabetes
Source: Diabetologia. 2014 Mar 1;57(5):891–901. doi: 10.1007/s00125-014-3196-x (PMC3980039; doi:10.1007/s00125-014-3196-x)
Supplement: Supplementary file 1 — (PDF 33 kb) [file 125_2014_3196_MOESM1_ESM.pdf]

ESM Table 1. Fasting and Postprandial Glucose Concentrations in Study 3[a]

	SITA 100 (n = 61)	CANA 300 (n = 74)
**FPG, mmol/l**		
Baseline	8.6 (2.6)	8.5 (1.9)
Week 52	8.0 (2.0)	6.9 (1.6)
Change from baseline	–0.57 (2.64)	–1.64 (2.05)
ΔLSM (95% CI)[b]	—	–1.13 (–0.54; –1.72)
p value[c]	—	0.0002
**$MPG_{0\text{-}3h}$, mmol/l**		
Baseline	14.4 (3.0)	14.2 (2.8)
Week 52	12.6 (2.8)	11.1 (2.2)
Change from baseline	–1.80 (2.90)	–3.13 (2.90)
ΔLSM (95% CI)[b]	—	–1.52 (–0.75; –2.30)
p value[c]	—	0.0002
**Maximum PG, mmol/l**		
Baseline	17.4 (3.4)	17.2 (3.6)
Week 52	15.4 (3.4)	13.7 (3.0)
Change from baseline	–2.0 (3.3)	-3.5 (3.7)
ΔLSM (95% CI)[b]	—	–1.71 (–2.69; –0.74)
p value[c]	—	0.0007

CANA 300, canagliflozin 300 mg; SITA 100, sitagliptin 100 mg.

[a]Data are mean (SD) unless otherwise indicated.

[b]ΔLSM is the SITA-subtracted LSM change from baseline.

[c]*p* value vs SITA at Week 52.
