# Supplementary material for: Canagliflozin, a sodium glucose co-transporter 2 inhibitor, improves model-based indices of beta cell function in patients with type 2 diabetes
Source: Diabetologia. 2014 Mar 1;57(5):891–901. doi: 10.1007/s00125-014-3196-x (PMC3980039; doi:10.1007/s00125-014-3196-x)
Supplement: Supplementary file 2 — (PDF 31 kb) [file 125_2014_3196_MOESM2_ESM.pdf]

ESM Table 2. Insulin Clearance Calculated from the MMTT[a]

	Study 1			Study 2			Study 3	
	PBO (n = 31)[a]	CANA 100 (n = 47)	CANA 300 (n = 42)	PBO (n = 33)	CANA 100 (n = 39)	CANA 300 (n = 33)	CANA 300 (n = 69)	SITA 100 (n = 58)
**Insulin clearance, l min^{-1} m^{-2}**								
Baseline	0.95 (0.43)	0.85 (0.34)	0.91 (0.38)	1.09 (0.44)	1.09 (0.34)	1.01 (0.38)	1.12 (0.41)	1.16 (0.50)
Endpoint[c]	0.91 (0.35)	1.00 (0.36)	1.06 (0.51)	1.07 (0.37)	1.16 (0.34)	1.24 (0.40)	1.44 (0.50)	1.15 (0.39)
ΔLSM (SE)[d]	—	0.17 (0.06)	0.19 (0.06)	—	0.09 (0.05)	0.24 (0.05)	0.31 (0.05)	0.01 (0.05)
p value[e]	—	0.002	0.001	—	0.07	<0.0001	<0.0001	—

CANA 100, canagliflozin 100 mg; CANA 300, canagliflozin 300 mg; PBO, placebo; SITA 100, sitagliptin 100 mg.

[a]Data are mean (SD) unless otherwise indicated.

[b]The number of patients with insulin clearance values is smaller than the number of patients with the other measures due to some patients having insufficient insulin measurements to perform the calculations.

[c]Week 26 for Studies 1 and 2; Week 52 for Study 3.

[d]ΔLSM is the PBO-subtracted LSM change from baseline for Studies 1 and 2 and the LSM change from baseline for Study 3.

[e]*p* value vs PBO for Studies 1 and 2, and vs SITA for Study 3.
